# Individual Capital Structure and Health Behaviors among Chinese Middle-Aged and Older Adults: A Cross-Sectional Analysis Using Bourdieu’s Theory of Capitals

**DOI:** 10.3390/ijerph17207369

**Published:** 2020-10-09

**Authors:** Peng Xu, Junfeng Jiang

**Affiliations:** 1 School of Health Sciences, Wuhan University, Wuhan 430071, China; pengxu@zuel.edu.cn; 2 Institute of Sociology, School of Philosophy, Zhongnan University of Economics and Law, Wuhan 430073, China

**Keywords:** Bourdieu, economic capital, social capital, cultural capital, health behavior

## Abstract

This study draws on Bourdieu’s theory of capitals to analyze the relative importance of economic, cultural, and social capital on health behaviors in Chinese middle-aged and older adults. Based on data from the China Family Panel Studies of 2016 (N = 15,147), we first harnessed a binary logistic regression model to discuss the associations between the three capitals and four types of health behaviors (i.e., physical exercise, smoking, binge drinking and stay-up). Using the sheaf coefficients technique, we then compared the relative effects of three of the capitals on health behaviors. The results suggest that cultural capital is the most influential one, which would significantly increase physical exercise and stay-up behaviors, and reduce smoking and binge drinking behaviors. Economic capital is also an important predictor, that may reduce smoking behavior but increase binge drinking and stay-up behaviors. Social capital has shown the least importance, although it would still be saliently associated with physical exercise, smoking and stay-up behaviors. In addition, some significant group disparities are also identified. This article is one of the first to explain health behavior inequalities through a Bourdieusian capital-based approach in Chinese contexts.

## 1. Introduction

With China’s economic growth and accelerated industrialization, profound changes have taken place in people’s lifestyles, as well as in the spectrum of diseases. As per the China Country Assessment Report on Ageing and Health, the main threat to population health has shifted away from maternal, child and acute infectious disorders to chronic non-communicable diseases, which is expected to be associated with at least a 40% increase in the burden of disease by the year 2030 [1]. Such changes are partly due to the aging of the demographic structure and the unhealthy dietary mode of Chinese residents [2,3,4]. In addition, the changing pattern of health-related behaviors has also played an important role. Specifically, the intensity of physical labor significantly decreased under the influence of China’s urbanization process, leading to an increasing number of people living a sedentary life without the necessary physical exercise [5,6]. To some extent, these changes in behavioral habits have given rise to the spread of chronic diseases, especially in middle-aged and older adults. Actually, empirical evidence has proven that health-related behaviors could exert cumulative long-term effects on individual morbidity and mortality [7,8,9,10]. Therefore, it is of great importance to identify the determinants of health-related behaviors (e.g., smoking, drinking, staying up, physical exercise), which would help to reduce the prevalence of chronic diseases.

In the existing literature, scholars often apply the Andersen’s health behavioral model to analyze the possible constraints on individuals’ health behaviors [11], which concentrates on three dimensions: predisposing demographic factors (e.g., age, gender), enabling factors (e.g., income, health insurance) and need factors (e.g., self-rated health status, health service need). Many studies have discussed the determinants of health behaviors based upon Andersen’s conceptual framework [12,13,14,15,16,17]. Nevertheless, most prior studies were concerned with medical treatment behaviors after illness, whilst limited attention was paid to the preventive behaviors, especially in Chinese contexts. Additionally, most studies focused on the general population, rather than the middle-aged and older adults who are at higher health risk. Furthermore, the relevant research mainly considered that health behaviors are either simply individualistic behavioral decisions, or are completely constrained by structural forces [18]. However, the field of health behaviors is not reducible to the intentions of individual agents or even to direct interactions between agents [19]. More precisely, from the perspective of medical sociology, any “choice” of health practice should be impacted by both organizing structure (i.e., socio-cultural environment) and individual agency factors [20,21].

Based on Bourdieu’s theory of capitals, the present paper tries to analyze the determinants of health behaviors among Chinese middle-aged and elderly people. In general, Bourdieu’s theoretical orientation places health behaviors within the family and social environment, with the particular emphasis that the components of individual capital structure, consisting of economic, cultural and social capital, could simultaneously impose complex effects on health behaviors [18,22,23,24]. In other words, different forms of capital possessed by individuals constitute the action resources that can be mobilized when there is a desire to improve one’s health. As a result, the combined characteristics of these resources may help to predict individuals’ health behavioral tendencies.

## 2. Theoretical Clarifications and Research Questions

Bourdieu’s capital theory argues that different capitals owned by individuals can determine their positions in the social stratification structure, and further influence the pattern of social behaviors. More specifically, there are three forms of capital, namely economic, social, and cultural capital. The acquisition and accumulation of different capitals is bound by individual socialization experience (i.e., the transition process from biological man to social man) [25]. However, the life events experienced in people’s life courses are not the same, and individuals will attain various life chances and be constrained by diverse social situations. Thus, there will be heterogeneity in the outcomes of socialization (e.g., the choice of health behaviors) among different social groups. In Bourdieu’s formulation, everyone is living in a particular “field”, which is a structured hierarchical space with its own operating rules and power relations. In such a social space, different actors occupy dominant or subordinate positions determined by the volume of each capital and the structure of capitals (i.e., relative amounts of different capitals) [26]. Thus, we argue that individual capital structure, shaped in the process of socialization, may influence one’s own dispositions, which correspond to particular “habitus” that reflect distinctive health behavioral tastes. In the following part, we will elaborate on the three forms of capital and their relationships with health behaviors.

Economic capital refers to the material wealth and financial assets owned by individuals or families. Personal/family income and assets are often used to measure the scale of individual’s economic resources. Prior studies reported significant associations between economic capital and health behaviors. For example, empirical evidence has shown that individuals with more income tend to smoke less [27], have lower probability of alcohol abuse [28], and enjoy a higher health-related quality of life [29]. As argued by Beck, in today’s risk society, much wealth is accumulated by the upper class, while risk aggregates mainly in the lower class. Thus, the capacity to cope with social risk will be distributed unequally among different social classes [30]. Beck’s viewpoints make a clear statement pertaining to the relationship between economic capital and health risk behaviors: in order to gain an advantageous position, individuals with more economic resources tend to care more about their health status, and are more likely to maintain a healthier lifestyle.

Social capital is also an important social determinant of health that has been widely discussed. Bourdieu defined social capital as the “aggregate of the actual or potential resources which are linked to the possession of a durable network of more or less institutionalized relationships of mutual acquaintance and recognition” [25]. Relevant studies have shown that social capital is significantly correlated with individual health status and health behaviors [31,32]. There are several possible explanations for the influence mechanism of social capital on health. Turner treated social capital consisting of individuals’ social relationships and social networks as “material investments in society”, which could play an important role in the maintenance of health status. Thus, the quantity of supportive networks and the quality of interpersonal connections are each capable of impacting on a variety of lifestyle factors, resulting in the fact that individuals with more valuable social networks are less likely to engage with social anomie behaviors, such as excessive drinking. In a sense, the notion of social capital has served as a critical tool to empirically discuss the relationship between social solidarity (e.g., social trust) and health outcomes in a given community [33]. Similarly, Suzuki and colleagues also found that the closer the social network members were to each other, the more likely they were to spread health norms and oppose unhealthy behaviors such as smoking and alcohol consumption [34]. In addition, health behaviors can be viewed as the results of internalized social norms, which may be affected by the surrounding environment. For example, a personal sleep schedule could be adjusted to the same time as when his/her neighbors turned off the lights. Thus, if members of a social network do not choose to stay up late, such a healthy habit may spread among network members [35]. Moreover, individuals living in a specific community will be influenced by the behavioral norms of surrounding people. That is to say, if neighbors regularly participate in physical activity (e.g., jogging, cycling), it may increase the likelihood of our participation in physical exercise [36,37].

Cultural capital is a special kind of capital in Bourdieu’s capital theory. It refers to the non-material things held and shared by people, which can serve as a symbolic representation of an individual’s socioeconomic status. It has three forms of existence: (1) The embodied state, which means the disposition acquired over the life course. For example, one’s behavioral habits can be formed through the process of parental instruction; (2) The institutionalized state, such as formal academic certificate; (3) The objectified state, which exists in the form of cultural objects (e.g., books in the home) [25]. Generally speaking, people with more cultural capital (e.g., the repertoire of cultural skills, verbal and nonverbal competencies) will participate in more advantageous health-related behaviors [18,38]. Prior studies revealed that compared to economic capital, cultural capital might be more effective in predicting health-related behaviors (e.g., smoking, diet) [39]. Gagné et al. also pointed out that both personal and parental education background were significant predictors of smoking behavior [40]. In this regard, cultural capital may help to better understand the mechanism by which social inequality in health behaviors develops.

In short, Bourdieu’s capital theory has been widely used to theoretically or empirically analyze health-related issues [18,38,41,42]. However, to the best of our knowledge, only a few studies have simultaneously analyzed the impacts of three forms of capital upon health status/health behaviors in European countries [22,23,24]. Relevant research in Chinese contexts is still limited. Admittedly, Bourdieu proposed his theory of capitals in France, but this does not preclude its potential applicability in other cultural contexts. In fact, the fundamental logic of health behavior ought to be similar across different countries: any "choice" of health practice can only be analyzed in relation to embodied dispositions (habitus) and the structure of capitals distributed unequally within the social field of health [18]. As argued by Schneider-Kamp, the construction of individual health has already become increasingly dependent on the availability and forms of personal disposable resources. As such, by embracing a Bourdieusian perspective, public health researchers are able to focus on the synergy of various forms of "health capital" and then understand "the social and cultural embeddedness of contemporary health practices" [43]. Following this line of thought, we insist that Bourdieu’s theoretical framework can be applied to delineate the heterogeneity of health in non-European contexts, such as Chinese society.

Even so, we still have to acknowledge that China has such social problems as remarkable regional variations, an uneven distribution of natural and human resources [44], and huge urban–rural health disparities [31]. Ichida and colleagues pointed out that the association between personal capitals and health outcome could vary according to socio-cultural circumstances [45]. As a result, the determinants of Chinese middle-aged and older adults’ health behaviors may be different from those of western societies. In light of this, the current study drew on Bourdieu’s theory of capitals to address the following questions:

(1) Do economic, social, and cultural capital have significant influence on health behaviors in Chinese middle-aged and older adults? Which form of capital matters more?
(2) Are the effects of individual capitals on different health behaviors consistent across urban/rural, gender, and age groups?

## 3. Materials and Methods

### 3.1. Data Source

This study is based on the China Family Panel Studies (CFPS) of 2016 jointly conducted by the China Social Science Survey Center of Peking University and the Survey and Research Center of the University of Michigan. CFPS is a nation-wide longitudinal survey started in 2010, and follow-up interviews are carried out every other year. A multiple-stage implicit stratification sampling design is used to collect the information of respondents aged 16 and above. CFPS mainly aims to provide a nationally representative sample that reflects Chinese family conditions, as well as the changes in Chinese residents’ education, ideology, health status and other aspects [46]. In our study, middle-aged and older adults aged 45 to 90 were selected. The 45-year-old is a highly recognized boundary between middle-aged and younger adults, while samples over 90 years old were excluded to reduce the adverse impact of elderly sample’s sparseness value on the accuracy of model analysis. After eliminating the data missing on capital and health behavior variables, 15,147 valid samples were finally obtained. 

### 3.2. Measurement

*Economic capital.* Compared with household wealth, personal income and occupation may become less important in the later years of life [47]. Therefore, we used “annual household income” and “household net assets” to measure the economic capital of middle-aged and older adults. “Annual household income” means the net income of all family members in the past year, namely gross household income after deducting all kinds of household expenses. “Household net assets” include net property, total financial assets, and the cash and savings of a certain household. Considering that the income and asset variables showed a right-skewed distribution, and that some families owned a negative asset value, we first sorted the samples by economic capital and subsequently divided them equally into 100 parts. Finally, we transformed them into two continuous variables ranging from 0 to 10 using the maximum difference normalization method.

*Cultural capital.* According to Bourdieu’s viewpoint [25], cultural capital includes three dimensions. “Institutionalized cultural capital” is measured by respondents’ current education [22,24], i.e., illiterate/uneducated = 0, primary school = 5, junior high school = 8, senior high/technical school = 11, 3-year college = 14, 4-year university = 15, masters = 18, doctor = 21. “Embodied cultural capital” is measured by the mother’s education, the father’s education and the number of books read in the past year. According to prior studies, embodied capital means a state embodied by behaviors and dispositions learned over the life course [22], and it refers to a kind of cultural ethos that is cultivated over a long period of time. From this point of view, parents’ education can be a macro family environment for people to develop this cultural ethos from childhood, and the reading habit can be an active way to develop this cultural ethos over the life course. Specifically, we first recoded parents’ education via “years of education”, then this was transformed into variables ranging from 0 to 10 using the maximum difference normalization method. For the right-skewed “number of books read” variable, a logarithmic transformation was firstly performed. Because there were zero values in the “number of books read” variable, the original values were multiplied by one before the log transformation, and similar strategies were also used in other places if no additional interpretation was shown. Then it was also transformed into a variable ranging from 0 to 10 using the maximum difference normalization method. Finally, the three variables were added and divided by three to yield the “embodied cultural capital” variable ranging from 0 to 10. “Objectified cultural capital” was measured by the number of books in the family [48], which was logarithmically transformed due to its right-skewed distribution.

*Social capital.* To measure social capital, we utilized general trust, special trust, and neighborhood cohesion, in line with prior relevant research [36,37]. Specifically, general trust was a binary variable that reflected the attitude towards the general population (not trust = 0, trust = 1). Special trust was concerned with the trust in five specific groups, including parents, neighbors, doctors, leading cadres and strangers. The responses for all five items were rated on a 0–10 scale with larger scores indicating a higher special trust level (Cronbach Alpha = 0.603). Neighborhood cohesion was measured by the following three questions: “What do you think of the neighborhood relationship?”, “Would a neighbor help you when you are in trouble?”, and “Do you like the community you live in?” The responses were rated on a 0-4 scale with higher scores indicating better neighborhood cohesion (Cronbach Alpha = 0.615). The above three items were added to create the neighborhood cohesion variable ranging from 0 to 12.

*Health behaviors.* Four measurements of health behavior were included in this study. Smoking behavior was a dichotomy variable (ex-smoker or current smoker = 1; non-smoker = 0). Drinking three times or more per week in the recent month was considered as binge drinking behavior (yes = 1; no = 0). Falling asleep between 23:00 o’clock and 17:00 o’clock the next day was treated as stay-up behavior (yes = 1; no = 0). It is worth mentioning that daytime sleepers usually have nightshifts or all-night behavior, so they were treated as having stay-up behavior. The physical exercise variable was measured through the question of whether the respondent had participated in physical activity in the past week (yes = 1; no = 0). 

*Control variables.* We controlled for gender, age, household registration, urban/rural residence, marital status, political status, work status, self-rated health (SRH), chronic disease and depression (see Table 1 for details). To be specific, the level of depression was measured by the CESD-8 scale (Cronbach Alpha = 0.799 in our sample). Eight depression-related emotional states were investigated, including being down in spirits, struggling to do things, insomnia, cheerfulness, loneliness, happiness, sadness, and hopelessness. The options were coded as “0 = almost no, 1 = in some cases, 2 = regularly, 3 = most of the time”. We summed eight items and yielded a depression variable ranging from 0 to 24, with higher scores indicating more serious depression. The descriptive statistics of our samples are summarized in Table 1.

### 3.3. Statistical Models

Depending on the outcome variables, we firstly utilized a binary logistic regression model to examine the relationship between individual capitals and health behaviors. In order to measure the relative contribution of three forms of capital, we chose to simultaneously add all the independent variables into the model. Specifically, the statistical model is as follows:

(1) $$\mathrm{logit}\left(\mathrm{Pr}\right)=\beta_{0}+\beta_{m}x_{m}+\beta_{n}x_{n}+\beta_{p}x_{p}+\beta_{q}x_{q}$$

In Equation (1), Pr represents the probability of each of the health behaviors. $\beta_{0}$ is the intercept term of the model. $x_{m}$, $x_{n}$ and $x_{p}$ stand for the variables of economic, cultural and social capital, respectively, while $x_{q}$ is a group of control factors.

Secondly, based on the above-mentioned logistic regression model, we further used the sheaf coefficients technique proposed by Heise [49]. Supposing that there are three latent variables indicating economic capital ($\eta_{1}$), cultural capital ($\eta_{2}$) and social capital ($\eta_{3}$), their corresponding relationships with three groups of independent variables are as follows:

(2) $$\eta_{1}=c_{1}+z_{m}x_{m}$$

(3) $$\eta_{2}=c_{2}+z_{n}x_{n}$$

(4) $$\eta_{3}=c_{3}+z_{p}x_{p}$$

Thus, Equation (1) can be rewritten into the following alternative form:

(5) $$\mathrm{logit}\left(\mathrm{Pr}\right)=\beta_{0}+\lambda_{1}\eta_{1}+\lambda_{2}\eta_{2}+\lambda_{3}\eta_{3}+\beta_{q}x_{q}$$

Equation (5) is the equivalent form of Equation (1) using the iterative method. $z_{m}$, $z_{n}$ and $z_{p}\;$ are three sets of post-estimated parameters. Three latent capital variables (i.e.,$\;\eta_{1},\;\eta_{2},\;\eta_{3}$) are standardized and their standard deviations equal 1. Hence the aggregate effects of the three latent capitals become comparable within the same equation. 

STATA version 15.0 was used to perform the binary logistic regression and “sheafcoef” command. *p* < 0.05 was considered to be statistically significant in this study, and data were weighted to yield a nationally representative result. 

## 4. Empirical Results

### 4.1. Results of Binary Logistic Regression Analysis

Table 2 shows that all three capitals were related to the health behaviors of middle-aged and older adults in China. With other variables controlled for, more family income was related to more binge drinking behavior; every additional unit of family income increased the probability of binge drinking by 3.6% (OR = 1.036, *p* < 0.05). Meanwhile, more family assets were negatively related to smoking behavior but positively associated with staying up behavior; every additional unit of family assets decreased the probability of smoking by 4.2% (OR = 0.958, *p* < 0.01) and increased the probability of staying up by 7.7% (OR = 1.077, *p* < 0.001).

Objectified and embodied cultural capitals were significantly associated with physical exercise and staying up behaviors in our samples of analysis. Every additional unit of objectified cultural capital increased the likelihood of physical exercise by 6.0% (OR = 1.060, *p* < 0.001), but it also increased the likelihood of staying up by 4.4% (OR = 1.044, *p* < 0.01). Besides, every additional unit of embodied cultural capital increased the odds of physical exercise by 14.4% (OR = 1.144, *p* < 0.001); for staying up behavior, this value was 8.5% (OR = 1.085, *p* < 0.05). By contrast, institutionalized cultural capital was evidently related to all outcome variables. Every additional unit of institutionalized cultural capital increased the probability of physical exercise by 5.5% (OR = 1.055, *p* < 0.001), and reduced the probability of smoking and binge drinking by 3.3% and 2.4%, respectively (OR = 0.967, *p* < 0.001; OR = 0.976, *p* < 0.01). Meanwhile, it also increased the odds of staying up by 2.1% (OR = 1.021, *p* < 0.01).

Investigating the contribution of social capital, we observed that it was significantly associated with physical exercise, smoking, and staying up behaviors. Compared with samples with low-level general trust, those with high-level general trust had a lower probability of staying up late (OR = 0.876, *p* < 0.05). Moreover, every additional unit of special trust decreased the probability of smoking by 1.3% (OR = 0.987, *p* < 0.01), while every additional unit of neighborhood cohesion increased the probability of physical exercise by 4.1% (OR = 1.041, *p* < 0.01) and decreased the probability of staying up by 3.3% (OR = 0.967, *p* < 0.05).

Finally, health behaviors were also related to other covariates. To be specific, males were more likely to have smoking and binge drinking behaviors than females. Urban residents had higher odds of participation in physical exercise than their rural counterparts, but the former were also more likely to stay up late. In addition, the odds of physical exercise increased with age, while the odds of smoking and staying up declined with age. Furthermore, there were also certain associations between medical needs variables (i.e., SRH, chronic disease and depression) and health behaviors.

### 4.2. Relative Importance of Three Capitals on Health Behaviors, Based on the Sheaf Coefficients Model

To examine which capital is more influential for health behaviors, this study further utilized the sheaf coefficients model to evaluate the relative effects of three capitals on health behaviors in Chinese middle-aged and older adults. In Table 3, Model 1 shows that cultural capital was the most important for physical exercise, because the relative effect of cultural capital on physical exercise was much larger than that of economic and social capital (0.365 > 0.016 and 0.365 > 0.095, respectively; χ2 = 62.29, *p* < 0.001). Models 2 and 3 show that cultural capital had a larger relative effect on smoking and binge drinking behaviors than economic and social capital; however, no statistically significant difference was observed among the three capitals. By contrast, Model 4 shows that economic and cultural capital had a larger effect on staying up behavior than social capital (χ2 = 9.67, *p* < 0.01); the effect of economic capital on staying up was 2.298 (0.216/0.094) times that of social capital, while the effect of cultural capital on staying up was 2.021 (0.190/0.094) times that of social capital.

**Figure 1:**
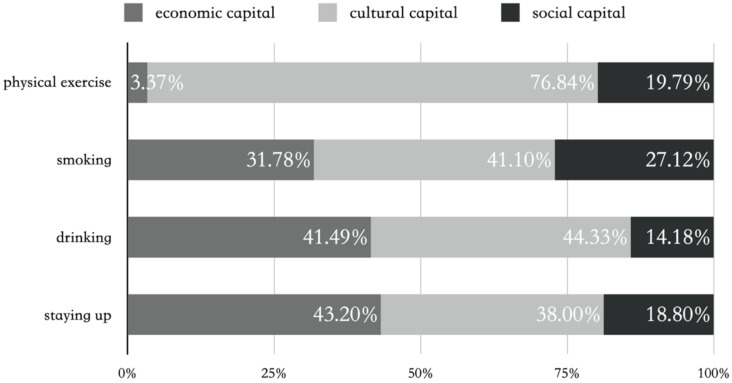
Relative contributions of economic, cultural and social capital to health behaviors.

We used Figure 1 to display three capitals’ relative effects on four health behaviors. It can be observed that cultural capital was the most important, as it explained 76.8% of the total variation in physical exercise caused by three capitals, and about 40.0% in other health behaviors. The results suggest that cultural capital accumulated during early socialization has a long-term impact on health behaviors later in life. In addition, economic capital also exerted a relatively large effect on all health behaviors except physical exercise. Nevertheless, social capital had a relatively small effect on almost all health behaviors. 

### 4.3. Group Disparities in Relationship between Capitals and Health Behaviors

Table 4 displays the results of group disparities, including sex, residence and age disparities, based on the sheaf coefficients model (results based on binary logistic regression models are provided in Appendix A, Table A1). Overall, the statistically significant results suggest the validity of Bourdieu’s capital theory in explaining the heterogeneity of health behaviors. Compared to the results shown in Table 3, group disparities in the relationship between capitals and physical exercise/staying up behaviors were identified, but intergroup differences in the relationships between capitals and smoking/drinking behaviors were not observed.

To be specific, Table 4 Panel A shows that, in both sexes, cultural capital had the largest association with physical exercise, followed by social capital and economic capital (χ2 = 21.50, *p* < 0.001 in males; χ2 = 47.09, *p* < 0.001 in females). Besides, cultural capital had a stronger relationship with physical exercise in females than in males. In addition, significant differences in the relationships between three capitals and staying up behavior were found in females (χ2 = 9.09, *p* < 0.05); furthermore, economic and cultural capital seemed more important than social capital in female stay-up behavior. However, no significant difference in the male group was observed.

Panel B indicates that the relationships between capitals and health behaviors were obviously different among urban and rural middle-aged and older adults. Cultural capital was highly related to physical exercise in both urban and rural samples (sheafcoef = 0.343, *p* < 0.001; sheafcoef = 0.385, *p* < 0.001), and social capital had a stronger association with physical exercise in rural samples (sheafcoef = 0.162, *p* < 0.001) than in their urban counterparts (sheafcoef = 0.070, *p* < 0.05); in addition, economic capital had almost no impact on physical exercise in both urban and rural areas.

Panel C suggests that the relationships between capitals and health behaviors were not consistent in different age groups. Cultural capital was still the most influential factor in both age groups, and its impact on all health behaviors declined with age. The relationships between the three capitals and staying up behavior were obviously different in the middle-aged group (χ2 = 15.59, *p* < 0.001), but the differences were nonsignificant in the older group. Moreover, in middle-aged adults, the effect sizes of economic and cultural capital on staying up behavior were 3.192 (0.249/0.078) and 3.090 (0.241/0.078) times that of social capital, respectively.

## 5. Discussion

Drawing on Bourdieu’s theory of capitals, this study attempted to analyze the relative importance of economic, cultural and social capital in terms of health behaviors in Chinese middle-aged and older adults. The results suggest that all three forms of capital could help to predict health behaviors in Chinese contexts; meanwhile, their effect sizes and directions seemed to be distinct, and some significant group disparities were also identified. These findings not only establish a direct link between individual capital structure and health behaviors, but also provide new evidence to explain the issue of health inequalities through a Bourdieusian capital-based approach.

Specifically, our findings confirmed that cultural and social capital obviously promoted physical exercise behavior, which was in line with previous studies [50,51]. Besides, among the three forms of capital, cultural capital was the most influential determinant of physical exercise. As a result, considering the connotation of cultural capital, we argue that participation in physical exercise is not only a personal choice, but an outcome of the socialization process by which durable behavioral norms are gradually embodied in the habitus of agents [18]. Similarly, social capital also played an important role in physical exercise behavior. In particular, we found that people living in higher neighborhood cohesion communities were more likely to be physically active; however, the positive effect of neighborhood social capital was significant mainly for rural residents. The possible explanation is that the rural community is like an “acquaintance society”, so rural residents tend to own more bonding social capital and be more familiar with people living in the same village. Thus, compared to the urban “stranger society”, behavioral norms are more easily identified within informal social networks in rural areas, which could further influence residents’ behavioral decisions [31].

In terms of smoking behavior, we observed that it was negatively related to almost all forms of capital in Chinese middle-aged and older adults. This finding is consistent with most prior studies and public perception [27,52]. In addition, the results also indicate significant group disparities in the relationship between capitals and smoking behavior. For instance, the reducing effect of institutionalized cultural capital (i.e., education level) on cigarette smoking was stronger in female, urban and middle-aged samples. This phenomenon may be due to inequalities in educational opportunity and quality among various social groups. Generally speaking, urban China has a better quality of education environment than rural China, and middle-aged adults tend to receive more education than older adults. Thus, those with better education should be more likely to be aware of the hazards of smoking behavior and develop the risk-aversion ability. Moreover, in light of the fact that females are generally less educated than males, the stronger inhibiting effect of cultural capital on smoking behavior among females may be ascribed to the higher marginal benefit. 

Our research also found that three forms of capital had different patterns of influence on drinking behavior. More precisely, economic capital increased the odds of alcoholism, while cultural capital did the opposite; however, social capital had no significant association with drinking behavior. Actually, in modern China, the traditional drinking culture has made drinking an important socializing behavior at the dinner table [53]. Since the direct economic cost of alcohol consumption is far higher than physical exercise, smoking and staying up behaviors, economic capital becomes a solid material foundation of drinking behavior, especially for those with higher odds of binge drinking, such as males and middle-aged adults. By contrast, the mainstream social norms are resistant to alcohol abuse, so a larger amount of cultural capital (e.g., education level that reflects internalized criteria of value) plays a positive role in reducing the risk of alcoholism [54]. As for social capital, it is embedded into personal social networks, thus the positive effects of interpersonal norms and the negative effects caused by drinking as a kind of socializing behavior are mixed, which may lead to social capital’s insignificant role. This finding is also consistent with other relevant studies [53,55].

As a kind of health risk behavior that accompanies the development of modern society, staying up is more common in the upper-middle class. Our study revealed that those with more economic/cultural capital tended to show higher odds of stay-up behavior. On the one hand, this finding could be directly explained by the fact that higher economic/cultural classes generally take more time to perform their jobs, which involve higher professional qualification. Thus, staying up late and working overtime seem to become the normal life of this group. On the other hand, we must clarify that stay-up behavior can also be seen as a means to gain status. In the modern labor market, some people like to share pictures of working overtime on their personal social networking sites, in order to purposively create the “looking-glass self”, i.e., let their friends know that they have a worthwhile job and they are hardworking. In this regard, stay-up behavior may help to show a sense of superiority compared to friends who have nothing to do, making oneself recognized as a “legitimate sufferer”. As such, status, based on perceptions of superiority, outcompetes concerns for health, leading to counterproductive health practices such as stay-up [43]. In addition, it should also be noted that cultural capital was not evidently associated with stay-up behavior in female and rural samples. The reasons for this may lie in the fact that “modernity” penetrates more deeply into urban and male adults, leading them to be more likely to stay up late. By contrast, social capital could reduce stay-up behavior in most subgroups except for females. In fact, more social capital, e.g., higher levels of neighborhood cohesion and general trust, may help to spread beneficial social norms such as “early to bed, early to rise”, which would positively influence individual health behaviors through personal social networks [35]. Furthermore, within the sex-differentiated labor market, it is obvious that males are more likely to develop the habit of staying up late due to job requirements, allowing social capital to play an important role in their stay-up behavior.

Lastly, our study further compared the relative contributions of three capitals to health behaviors using the sheaf coefficients technique. It is observed that cultural capital was the most important one, especially as regards physical exercise behavior. As argued above, cultural capital reflects the role of long-term socialization pertaining to the internalization of social behavioral norms, and such norms can further determine the dispositions of health behaviors [39,40]. Economic capital showed the second strongest association with health behaviors among the three capitals. Besides, considering it may increase the probability of health risk behaviors (e.g., drinking and staying up), attention should be paid to the negative effect of material wealth on health behaviors. In contrast, social capital had the least explanatory power. This finding suggests that the effect of social capital on health outcome may be over-estimated [56]. As a result, we should be scrupulous when discussing the role of social capital in the future.

There are still some limitations to this study. First, cross-sectional data were used in this study. Thus, the causal relationship between different capitals and health behaviors cannot be clearly inferred. A longitudinal design is needed to confirm our findings’ robustness. Second, social capital was measured based upon self-reported information in our analysis. More comprehensive social capital indicators, such as social participation and interaction, need to be introduced in future studies. Third, the measure of drinking behavior is not rigorous, because we cannot know what kind of liquor individuals drink, or how much liquor they take in each time, based on the existing survey data from CFPS2016. Finally, the composition of capitals (e.g., high in sociocultural capital and low in economic capital) is specifically mentioned in Bourdieu’s capital theory, which may influence the mode of health behavior [22,25]. Nevertheless, these ideas were not tested in the present study. Therefore, future research should fill in this gap by considering the impacts of the capitals’ composition.

## 6. Conclusions

For years, research on the determinants of health behaviors had been limited to micro-level characteristics. Recently, public health scholars have paid more attention to the broader socio-cultural environment wherein health behaviors occur [37,57]. Our study further bolsters the importance of applying the Bourdieusian capital-based approach to explain inequalities in health behaviors. The results suggest that the ownership of economic, cultural and social capital can influence individuals’ health practices; however, the relative contribution of the three capitals seems to be heterogeneous. Cultural capital is the most important one for health behaviors, which would significantly increase physical exercise and stay-up behaviors, and reduce smoking and binge drinking behaviors. Economic capital is also an important predictor, that may reduce smoking behavior but increase binge drinking and stay-up behaviors. Social capital has shown the least importance, although it can still be saliently associated with physical exercise, smoking and stay-up behaviors. In sum, as demonstrated in our empirical evidence, the association between individual capital structure and health behaviors is very complex, rather than being a simple linear relationship. The above-mentioned findings would help us better interpret the social determinants of health behaviors and promote our understanding of the health inequality problem in China. 

In future studies, since Bourdieu’s analytic framework is in essence “relational”, we need to relationally penetrate and explain how a structure of interdependent positions or the distribution of capitals within the social field of health affects patterns of health practice [18]. Moreover, the importance of social distinction in framing both objective and symbolic forms of capital described by Bourdieu also offers enlightening clues to aid in understanding adults’ health lifestyles in modern times. In this sense, investigating the heterogeneity of health behaviors embedded into the lives of people around the world through each form of accrued and accruing capitals—together with their symbolic representations and their patterns of composition—should be of great value to health behavior research [26]. Finally, in policy terms, the findings suggest that in order to further improve public health, relevant authorities should strike a balance between "caring and empowering" [58]. That is to say, on the one hand, the government needs to take the initiative to provide people with health behavior guidelines or public physical exercise facilities; on the other hand, encouraging middle-aged and older adults to develop their own capital-based resources is also necessary. Meanwhile, policy interventions need to take into account the inequalities in the possession and distribution of capital resources in order to guide people to live a healthier lifestyle.

## Figures and Tables

**Table 1:** Descriptive statistics of samples.

Variables	Coding	Mean	SD
**Economic Capital**			
Annual household income (CNY)	continuous variable	76,580	228,966
Household net assets (CNY)	continuous variable	578,059	1,742,381
**Cultural Capital**			
Embodied state	continuous variable	0.646	0.912
Objectified state	continuous variable	61.944	541.685
Institutionalized state	continuous variable	4.619	4.323
**Social Capital**			
General trust	not trust = 0; trust = 1	0.541	0.498
Special trust	continuous variable	29.887	6.933
Neighborhood cohesion	continuous variable	9.462	1.889
**Health Behaviors**			
Physical exercise	no = 0; yes = 1	0.422	0.494
Smoking	non-smoker = 0; ex-smoker or current smoker = 1	0.297	0.457
Drinking	no = 0; yes = 1	0.173	0.378
Stay-up	no = 0; yes = 1	0.203	0.402
**Control Variables**			
Sex	female = 0; male = 1	0.492	0.500
Age	continuous variable	59.865	10.561
Residence	rural = 0; urban = 1	0.465	0.499
Household registration	agricultural registration = 0; non-agricultural registration = 1	0.279	0.448
Marital status	not married = 0; married = 1	0.849	0.358
Political status	member of Communist Party of China (no = 0; yes = 1)	0.094	0.292
Work status	no job = 0; employment = 1	0.670	0.470
Self-rated health (SRH)	very poor/poor/fair = 0; good/very good = 1	0.526	0.499
Chronic disease	no = 0; yes = 1	0.245	0.430
Depression	continuous variable	5.487	4.412

Notes: CNY = Chinese Yuan; SD = Standard Deviation; Annual household income, household net assets, and the objectified state of cultural capital were reported in the original value.

**Table 2:** Associations between capitals and health behaviors in Chinese middle-aged and older adults.

	Model 1: Physical Exercise	Model 2: Smoking	Model 3: Drinking	Model 4: Staying Up
	OR (95% CI)	OR (95% CI)	OR (95% CI)	OR (95% CI)
**Economic capital**				
Family income	1.003	1.005	1.036 *	1.001
	(0.981, 1.024)	(0.980, 1.032)	(1.008, 1.065)	(0.977, 1.027)
Family assets	1.004	0.958 **	1.008	1.077 ***
	(0.983, 1.025)	(0.933, 0.983)	(0.981, 1.036)	(1.051, 1.104)
**Cultural capital**				
Objectified capital	1.060 ***	0.996	0.999	1.044 **
	(1.034, 1.086)	(0.966, 1.027)	(0.969, 1.030)	(1.015, 1.074)
Embodied capital	1.144 ***	0.996	0.967	1.085*
	(1.078, 1.214)	(0.930, 1.066)	(0.899, 1.040)	(1.018, 1.156)
Institutionalized capital	1.055 ***	0.967 ***	0.976 **	1.021 **
	(1.040, 1.070)	(0.951, 0.984)	(0.959, 0.993)	(1.005, 1.037)
**Social capital**				
General trust	0.926	0.950	0.963	0.876 *
	(0.836, 1.024)	(0.837, 1.077)	(0.846, 1.096)	(0.778, 0.986)
Special trust	1.007	0.987 **	0.995	1.001
	(0.999, 1.014)	(0.977, 0.997)	(0.985, 1.006)	(0.992, 1.010)
Neighborhood cohesion	1.041 **	1.031	1.012	0.967 *
	(1.012, 1.070)	(0.996, 1.067)	(0.975, 1.050)	(0.936, 1.000)
**Control variables**				
Sex	0.920	22.126 ***	10.208 ***	0.976
	(0.829, 1.021)	(18.418, 26.579)	(8.558, 12.177)	(0.866, 1.101)
Age	1.013 ***	0.983 ***	0.996	0.966 ***
	(1.006, 1.019)	(0.975, 0.991)	(0.989, 1.004)	(0.959, 0.974)
Residence	1.397 ***	1.037	0.999	1.683 ***
	(1.262, 1.547)	(0.913, 1.177)	(0.875, 1.140)	(1.485, 1.908)
Hukou	1.327 ***	1.023	0.917 *	1.356 ***
	(1.243, 1.416)	(0.940, 1.114)	(0.841, 1.000)	(1.260, 1.459)
Marital status	1.094	0.744 *	0.859	1.079
	(0.928, 1.291)	(0.591, 0.937)	(0.679, 1.086)	(0.879, 1.324)
Work status	0.616 ***	1.356 ***	1.414 ***	1.012
	(0.545, 0.697)	(1.148, 1.602)	(1.188, 1.682)	(0.876, 1.169)
Political status	1.591 ***	0.908	1.025	1.180
	(1.330, 1.903)	(0.751, 1.096)	(0.845, 1.245)	(0.978, 1.424)
SRH	1.158 **	1.172 *	1.191 *	1.123
	(1.041, 1.288)	(1.027, 1.339)	(1.037, 1.368)	(0.992, 1.271)
Chronic disease	1.324 ***	0.683 ***	0.678 ***	1.236 **
	(1.173, 1.496)	(0.583, 0.800)	(0.572, 0.804)	(1.076, 1.419)
Depression	0.976 ***	1.015	0.983	1.021 *
	(0.964, 0.989)	(0.998, 1.032)	(0.965, 1.001)	(1.005, 1.037)
Intercept	0.083 ***	0.204 ***	0.064 ***	0.478 *
	(0.045, 0.151)	(0.098, 0.426)	(0.030, 0.139)	(0.235, 0.972)
Pseudo_R^2^	0.104	0.263	0.166	0.084
n	15,144	15,147	15,147	15,142

Notes: *** *p* < 0.001, ** *p* < 0.01, * *p* < 0.05; OR = Odds Ratio; CI = confidence interval; All four models were weighted, and the 95% CI is shown in parentheses.

**Table 3:** Relative effects of three capitals on health behaviors in Chinese middle-aged and older adults.

	Model 1: Physical Exercise	Model 2: Smoking	Model 3: Drinking	Model 4: Staying Up
	Sheafcoef (95% CI)	Sheafcoef (95% CI)	Sheafcoef (95% CI)	Sheafcoef (95% CI)
Economic capital	0.016	0.116 **	0.117 **	0.216 ***
	(−0.040, 0.073)	(0.049, 0.183)	(0.044, 0.190)	(0.152, 0.280)
Cultural capital	0.365 ***	0.150 ***	0.125 **	0.190 ***
	(0.303, 0.427)	(0.078, 0.222)	(0.048, 0.202)	(0.123, 0.257)
Social capital	0.095 ***	0.099 **	0.040	0.094 **
	(0.045, 0.145)	(0.038, 0.161)	(−0.025, 0.105)	(0.037, 0.152)
Control variables	√	√	√	√
Intercept	−2.491 ***	−1.588 ***	−2.746 ***	−0.739 *
	(−3.093, −1.889)	(−2.323, −0.852)	(−3.521, −1.971)	(−1.450, −0.028)
χ2	62.29 ***	1.09	3.45	9.67 **
Pseudo_R^2^	0.104	0.263	0.166	0.084
n	15,144	15,147	15,147	15,142

Notes: *** *p* < 0.001, ** *p* < 0.01, * *p* < 0.05; CI = Confidence interval; All four models were weighted, and the 95% CI is shown in parentheses.

**Table 4:** Group disparities in the relationships between capitals and health behaviors.

	Model 1: Physical Exercise		Model 2: Smoking		Model 3: Drinking		Model 4: Staying Up	
	Sheafcoef (95% CI)		Sheafcoef (95% CI)		Sheafcoef (95% CI)		Sheafcoef (95% CI)	
Panel A: sex	male	female	male	female	male	female	male	female
Economic Capital	0.061	0.044	0.103 **	0.181	0.115 **	0.184	0.224 ***	0.212 ***
	(−0.017, 0.140)	(−0.029, 0.116)	(0.032, 0.174)	(−0.011, 0.373)	(0.038, 0.192)	(−0.012, 0.380)	(0.131, 0.317)	(0.124, 0.300)
Cultural Capital	0.344 ***	0.391 ***	0.127 **	0.370 ***	0.125 **	0.215	0.208 ***	0.171 ***
	(0.256, 0.431)	(0.304, 0.477)	(0.049, 0.205)	(0.164, 0.576)	(0.045, 0.204)	(−0.008, 0.439)	(0.115, 0.301)	(0.076, 0.265)
Social Capital	0.113 **	0.081 *	0.110 **	0.202 *	0.065	0.120	0.152 ***	0.046
	(0.040, 0.186)	(0.012, 0.150)	(0.045, 0.175)	(0.049, 0.354)	(−0.004, 0.134)	(−0.044, 0.283)	(0.072, 0.232)	(−0.034, 0.126)
Control Variables	√	√	√	√	√	√	√	√
χ2	21.50 ***	47.09 ***	0.19	1.88	1.41	0.45	1.62	9.09 *
Pseudo_R^2^	0.123	0.087	0.043	0.057	0.028	0.027	0.096	0.076
n	7598	7546	7599	7548	7599	7548	7596	7546
**Panel B: residence**	**urban**	**rural**	**urban**	**rural**	**urban**	**rural**	**urban**	**rural**
Economic Capital	0.029	0.025	0.130 **	0.068	0.120 *	0.111 **	0.211 ***	0.242 ***
	(−0.047, 0.105)	(−0.037, 0.087)	(0.044, 0.216)	(−0.011, 0.147)	(0.018, 0.223)	(0.028, 0.193)	(0.134, 0.289)	(0.153, 0.332)
Cultural Capital	0.343 ***	0.385 ***	0.199 ***	0.104 **	0.127 *	0.126 **	0.213 ***	0.152 **
	(0.259, 0.427)	(0.314, 0.455)	(0.098, 0.299)	(0.028, 0.181)	(0.019, 0.234)	(0.044, 0.207)	(0.128, 0.299)	(0.066, 0.238)
Social Capital	0.070 *	0.162 ***	0.092 *	0.128 **	0.088	0.067	0.104 **	0.094 *
	(0.004, 0.137)	(0.095, 0.229)	(0.008, 0.176)	(0.054, 0.203)	(−0.003, 0.179)	(−0.014, 0.149)	(0.033, 0.175)	(0.012, 0.175)
Control Variables	√	√	√	√	√	√	√	√
χ2	30.74 ***	58.57 ***	2.47	1.29	0.34	1.04	5.84 *	5.90 *
Pseudo_R^2^	0.092	0.049	0.243	0.307	0.161	0.180	0.079	0.028
n	6988	8156	6988	8159	6988	8159	6986	8156
**Panel C: age**	**45** **–** **59**	**60** **–** **90**	**45** **–** **59**	**60** **–** **90**	**45** **–** **59**	**60** **–** **90**	**45** **–** **59**	**60** **–** **90**
Economic Capital	0.045	0.019	0.112 **	0.107 *	0.166 ***	0.086	0.249 ***	0.199 ***
	(−0.020, 0.110)	(−0.057, 0.095)	(0.032, 0.192)	(0.002, 0.212)	(0.082, 0.250)	(−0.033, 0.205)	(0.176, 0.322)	(0.091, 0.307)
Cultural Capital	0.406 ***	0.310 ***	0.162 **	0.106 *	0.139 **	0.110	0.241 ***	0.215 ***
	(0.332, 0.480)	(0.215, 0.405)	(0.071, 0.254)	(0.003, 0.209)	(0.052, 0.227)	(−0.005, 0.225)	(0.163, 0.318)	(0.115, 0.315)
Social Capital	0.093 **	0.124 **	0.099 **	0.125 **	0.041	0.103 *	0.078 *	0.134 **
	(0.030, 0.157)	(0.048, 0.200)	(0.025, 0.173)	(0.031, 0.219)	(−0.036, 0.118)	(0.000, 0.208)	(0.009, 0.147)	(0.040, 0.228)
Control Variables	√	√	√	√	√	√	√	√
χ2	53.82 ***	20.33 ***	1.14	0.10	4.93	0.11	15.59 ***	1.56
Pseudo_R^2^	0.092	0.110	0.330	0.206	0.206	0.140	0.083	0.062
n	8335	6809	8337	6810	8337	6810	8336	6806

Notes: *** *p* < 0.001, ** *p* < 0.01, * *p* < 0.05; CI = Confidence interval; All models were weighted, and the 95% CI is shown parentheses.

## Appendix A

**Table A1:** Group disparities in associations between capitals and health behaviors in Chinese middle-aged and older adults, based on binary logistic regression analysis.

Panel A: Sex	Exercise: OR		Smoking: OR		Drinking: OR		Staying up: OR	
	Male	Female	Male	Female	Male	Female	Male	Female
Family income	1.022	0.982	1.010	0.988	1.041 **	1.014	1.007	0.997
Family assets	0.998	1.009	0.960 **	0.946	0.999	1.057	1.077 ***	1.078 ***
Objectified capital	1.060 **	1.061 **	0.995	0.980	1.008	0.932	1.044 *	1.044 *
Embodied capital	1.162 ***	1.117 *	0.999	0.969	0.964	0.957	1.073	1.099 *
Institutionalized capital	1.047 ***	1.067 ***	0.972 **	0.924 **	0.974 **	0.978	1.029 *	1.014
General trust	0.914	0.937	0.973	0.821	0.972	0.909	0.843 *	0.917
Special trust	1.008	1.005	0.984 **	0.993	0.991	1.011	0.999	1.004
Neighborhood cohesion	1.047 *	1.036	1.011	1.108 *	1.023	0.946	0.942 *	0.993
Control variables	√	√	√	√	√	√	√	√
Pseudo_R^2^	0.123	0.087	0.043	0.057	0.028	0.027	0.096	0.076
n	7598	7546	7599	7548	7599	7548	7596	7546
**Panel B: Residence**	**urban**	**rural**	**urban**	**rural**	**urban**	**rural**	**urban**	**rural**
Family income	1.010	0.990	1.012	0.998	1.039 *	1.033 *	0.989	1.042 *
Family assets	1.001	1.007	0.951 **	0.975	1.007	1.012	1.082 ***	1.065 **
Objectified capital	1.064 ***	1.052 **	0.991	1.014	1.001	1.000	1.034	1.077 **
Embodied capital	1.142 **	1.150 **	0.976	1.062	0.944	1.031	1.108 **	1.006
Institutionalized capital	1.043 ***	1.083 ***	0.961 **	0.973 *	0.980	0.967 **	1.024 *	1.010
General trust	0.927	0.938	0.923	1.000	0.919	1.043	0.837 *	0.972
Special trust	1.006	1.008	0.990	0.981 **	0.992	1.002	1.002	0.999
Neighborhood cohesion	1.026	1.079 ***	1.036	1.019	1.039	0.964	0.973	0.953 *
Control variables	√	√	√	√	√	√	√	√
Pseudo_R^2^	0.092	0.049	0.243	0.307	0.161	0.180	0.079	0.028
n	6988	8156	6988	8159	6988	8159	6986	8156
**Panel C: Age**	**45–59**	**60–90**	**45–59**	**60–90**	**45–59**	**60–90**	**45–59**	**60–90**
Family income	1.000	1.008	1.018	1.005	1.059 **	1.023	1.018	0.989
Family assets	1.016	0.994	0.954 **	0.962 *	1.006	1.009	1.083 ***	1.076 ***
Objectified capital	1.053 **	1.063 **	0.998	1.000	1.022	0.979	1.082 ***	1.012
Embodied capital	1.210 ***	1.035	1.027	0.975	0.928	1.042	1.067	1.186 **
Institutionalized capital	1.057 ***	1.055 ***	0.960 **	0.978	0.977 *	0.977	1.022 *	1.027 *
General trust	0.990	0.888	0.961	0.929	0.958	0.963	0.940	0.791 *
Special trust	0.998	1.015 *	0.986 *	0.985 *	1.005	0.986	0.998	1.001
Neighborhood cohesion	1.054 **	1.028	1.009	1.043	0.983	1.034	0.967	0.970
Control variables	√	√	√	√	√	√	√	√
Pseudo_R^2^	0.092	0.110	0.330	0.206	0.206	0.140	0.083	0.062
n	8335	6809	8337	6810	8337	6810	8336	6806

Notes: *** *p* < 0.001, ** *p* < 0.01, * *p* < 0.05; OR = Odds Ratio; All four models were weighted, and the 95% confidence intervals were not reported in order to save space.
